# Assessing Exposures to Household Air Pollution in Public Health Research and Program Evaluation

**DOI:** 10.1007/s10393-014-0990-3

**Published:** 2014-11-08

**Authors:** Amanda L. Northcross, Nina Hwang, Kalpana Balakrishnan, Sumi Mehta

**Affiliations:** 1 Department of Environmental and Occupational Health, George Washington University Milken Institute School of Public Health, 950 New Hampshire Ave 7th Floor, 20057 Washington, DC USA; 2 Department of Environmental Health Engineering, Sri Ramachandra University, Chennai, India; 3 Global Alliance for Cleancook Stoves, Washington, DC USA

**Keywords:** household air pollution, exposure assessment, cookstoves

## Abstract

Exposure to smoke from the use of solid fuels and inefficient stoves for cooking and heating is responsible for approximately 4 million premature deaths yearly. As increasing investments are made to tackle this important public health issue, there is a need for identifying and providing guidance on best practices for exposure and stove performance monitoring, particularly for public health research and evaluation studies. This paper, which builds upon the discussion at an expert consultation on exposure assessment convened by the Global Alliance for Clean Cookstoves, the Centers for Disease Control and Prevention, and PATH in late 2012, aims to provide general guidance on what to monitor, who and where to monitor, and how to monitor household air pollution exposures. In addition, we summarize information about commercially available monitoring equipment and the technical properties of these monitors most important for household air pollution exposure assessment. The target audience includes epidemiologists conducting health studies and program evaluators aiming to quantify changes in exposures to estimate the potential health benefits of cookstoves intervention projects.

## Introduction

Exposure to household air pollution (HAP) causes over 4 million deaths each year (Lim et al. 2012; World Health Organization 2014). Nearly, all of these deaths occur in low and middle income countries, making HAP one of the leading risk factors for health on a global scale (Lim et al. 2012). While the magnitude of the problem is now well acknowledged, to date most epidemiologic studies have suffered from the use of simplistic approaches to estimating exposures, often due to limited resources. For example, until very recently, most studies have included only binary classifications of exposure. Clean cookstoves, which the International Standards Organization (ISO) defines as those with lower emissions, are widely considered as an effective intervention for exposure to HAP. However, the evaluation of cookstove dissemination projects has included little to no quantitative assessment of changes in HAP exposures, much less an assessment of health improvements. This has severely hindered the cooking sector’s ability to define just how ‘clean’ is clean enough to achieve health benefits.

The Global Alliance for Clean Cookstoves, Centers for Disease Control and Prevention, and Program for Appropriate Technology in Health (PATH) organized a technical workshop in late 2012 on harmonizing approaches to exposure assessment. The purpose of the workshop was to

- Discuss approaches to assessing household and ambient personal exposures to PM_2.5_ and CO for health research and program evaluation;
- Develop a harmonized framework to standardize methods for measurement and monitoring so that comparable data can be collected in health research studies and program evaluations
- Identify key technological barriers to achieving high-quality field measurement of personal exposure, and discuss work in progress and/or necessary to address these barriers; and
- Identify key product attributes and requirements to achieve high-quality field measurements for personal exposure.

Stemming from discussions and conclusions made during the workshop, the purpose of this paper is to provide practical technical guidance on assessing exposures to HAP for health research and program evaluation. It should be noted that the paper should not be considered a traditional meeting report for the following two reasons:

1. Several of the workshop participants were members of the Exposure Assessment and Biomarkers Working Group at the May 2011 NIH meeting ‘Health Burden of Indoor Air Pollution on Women and Children in Developing Countries’, and co-authored a paper which defined research priorities for exposure assessment (Clark et al. 2013), and included a detailed discussion of many of the topics discussed in this workshop.
2. While the initial aim of the workshop was to discuss recommended approaches to promote harmonizing exposure assessment of HAP, workshop participants ultimately deemed the use of a standardized approach to be an unnecessary goal. Greater priority was placed on promoting the quality of exposure assessment methods applied in epidemiology and evaluation projects by advocating for the inclusion of dedicated exposure experts within interdisciplinary study teams.

As such, this paper is intended to provide public health researchers with general guidance on what to monitor, who and where to monitor, and how to monitor in field studies assessing HAP exposures. It should be noted, therefore, that the technical information addressed here is not meant to serve as a ‘recipe’ for epidemiologists interested in conducting research on HAP and health. Rather, it is intended to highlight some key practical information to consider in order to enable a productive dialog between the epidemiologist and the dedicated exposure scientist within a study’s interdisciplinary team. This paper also provides cookstove program evaluators with a starting point to understand how to assess HAP exposures from cookstoves. Moreover, the paper also raises the critical issue of the availability of necessary human resources and technical capacity to execute detailed exposure assessments in resource-constrained settings.

## Why Monitor?

Exposures to household air pollution are monitored for several reasons. Environmental health scientists are keenly focused on determining an exposure–response relationship with HAP and the cascade of known health effects stemming from exposures. Clark et al. (2013) summarized research priorities aimed at reaching this goal, which are focused on reducing uncertainty in exposure assessment and reducing measurement error due to unexplained variability. Most recently, the National Institute for Environmental Health Sciences hosted a workshop on ‘Assessing Exposures and Health Effects Related to Indoor Biomass Fuel Burning’, which included a detailed discussion of the need for additional lab and field-based evidence on the exposures resulting from the wide range of ‘cleaner’ cooking technologies, including cleaner stoves and fuels. In addition to directly assessing the impact of HAP on health, studies and projects have also focused on behaviors which lead to adopting cookstoves (Mukhopadhyay et al. 2012; Shankar et al. 2014), evaluating changes in exposures due to a cookstove intervention within scientific research studies as well as development-focused programs (Sinton et al. 2004; Venkataraman et al. 2010), cookstove intervention feasibility studies (Pine et al. 2011), as well as assessing baseline exposures to HAP (Clark et al. 2011; Commodore et al. 2013).

## What to Monitor?

Household air pollution is comprised of hundreds of compounds, many of which can be toxic or carcinogenic (Naeher et al. 2007). However, carbon monoxide (CO) and particulate matter with an aerodynamic size less than or equal to 2.5 μm (PM_2.5_) are commonly the only two pollutants measured to assess exposure to cookstove smoke. Biological explanations for health effects associated with exposures to CO and PM_2.5_ have been proposed, and both pollutants can be measured without the need for expensive chemical composition analysis. Although there is growing evidence that the source and composition of particulate matter may affect toxicity and health outcomes, global standards are based on the mass-based measurement of particulate matter, rather than composition of particulate matter. Guidelines have been set by the World Health Organization (2006) for mass concentrations of PM_2.5_ and CO (Table 1). Due to the availability of international guidelines and the focus of the scientific community on these two pollutants, this paper discuses monitoring CO and PM_2.5_ concentrations. The exclusion of other pollutants from this discussion, including volatile and semi-volatile organic compounds and ultra-fine particles, should not be interpreted as unimportant for future studies.

**Table 1:** WHO Guidelines for CO and PM_2.5_

Averaging time	CO mg/m^3^	PM_2.5_ μg/m^3^	PM_10_ μg/m^3^
Annual	–	10	20
24 h	–	25	50
15 min	100	–	–
1 h	35	–	–
8 h	10	–	–
24 h	7	–	–

### PM_2.5_

Fine particulate matter is composed of particles with an aerodynamic diameter less than 2.5 μm (PM_2.5_). When inhaled, fine particles can traverse deep into the lungs to deposit in the alveoli or cross into the blood stream where they create significant health impacts (World Health Organization 2006). PM_2.5_ exposure may cause pulmonary and systemic inflammation (Brook et al. 2010) and exacerbate existing diseases in the respiratory tract, such as asthma (Yu et al. 2000). Health effects associated with exposure to PM_2.5_ also include cardiovascular disease (Brook et al. 2010), impaired pulmonary function (Aunan et al. 2013), reduced immune function (Banerjee et al. 2012), and cognitive impacts (Dix-Cooper et al. 2012).

PM_2.5_ exposures to HAP can be measured using either gravimetric sampling or optical direct-reading monitors. Gravimetric sampling requires a sampling pump to draw air through a size-selective inlet (cyclone or impactor) designed to collect particles of a specific size or less. The size-selected particles are captured by a filter weighed before and after sampling allowing for the calculation of the particle mass collected. The resultant measurement of gravimetric monitoring is an integrated average over the sampling time. Gravimetric sampling is treated as the gold standard for measuring PM_2.5_ mass concentration because it directly measures mass, rather than a proxy for mass measured by optical direct-reading monitors which rely on light scattering measurement techniques. In addition, gravimetric samples may be further analyzed to assess the chemical composition of particles, including their potential climate impacts, and to provide information on the major sources of PM. Challenges associated with gravimetric sampling include the need for careful filter handling and an expensive analytical balance for weighing the filters that is maintained within a temperature, and humidity, controlled weighing lab. While gravimetric sampling traditionally required the use of relatively heavy and noisy monitors, recent technologic developments have resulted in the ability to conduct gravimetric sampling with less discomfort to study participants.

Optical monitors, which rely on light scattering to measure particle concentrations, provide data at a higher time resolution (most have a minimum one-minute time resolution) and require fewer pieces of equipment than gravimetric sampling. Particles can be sampled either passively or actively. As the particles pass through a light source in the monitor, light is scattered and read by a photo-detector. The signal from the photo-detector is used to determine the particle concentration. Optical instruments can be calibrated to a specific type of particle to provide an estimate of particle mass concentration. Since particles from different sources have different light scattering properties, adjusting values from an optical monitor with a gravimetric mass concentration measurement collected concurrently results in more accurate measurements. Light scattering methods estimate particle sizes, while a size-selective inlet provides a more exact method to measure particles of a specified size. Table 3 summarizes both light scattering monitors and PM measurement systems, which rely on a physical size cut.

### CO

Carbon monoxide is a colorless, odorless, and tasteless gas produced through incomplete combustion. Acute exposure to high levels of CO (>100 ppm) can cause headaches, dizziness, vomiting, and loss of consciousness (CDC 2013). The health effects of chronic exposure to low levels of CO are unclear. Studies have suggested that maternal CO exposure increases the incidence of low birth weight and perinatal deaths (Astrup et al. 1972). CO exposure can either be measured as an airborne concentration or as biomarkers in exhaled breath or blood samples.

Airborne measurements of CO concentrations are conducted using colorimetric tubes or electrochemical cells in HAP studies (Table 2). These two methods are relatively inexpensive and provide accurate measurements (when instruments are well calibrated), but are limited by the cross-sensitivity of the detection method with other pollutants, and by limits of detection, which can be as high as 2 ppm.

**Table 2:** Summary of Commercially Available CO Monitors

Name	Max battery life (h)	Battery type	Active/passive	Integrated/continuous	Data logging	Zero correctable	Detection method	Measurement range/resolution (ppm)
Gasbadge Pro	2,600	3 V, CR2 Li	Passive	Continuous	Yes	Yes	Electrochemical sensor	0–1,500/2
Lascar EL-USB-CO300	2,160	2 AA	Passive	Continuous	Yes	No	Electrochemical sensor	0–300/2
Dräger pac5000	>10,400	Rechargeable Li	Passive	Continuous	Yes	Yes	Electrochemical sensor	0–500/2
Dräger pac7000	>10,400	Rechargeable Li	Passive	Continuous	Yes	Yes	Electrochemical sensor	0–1999/2
Gastec Passive Diffusion tubes 1D	Measuring time: 0.5–48 h	none	Passive	Integrated	No		Color Dosimetry	LOD: 2 ppm (10 h) 1.4–2,000
Gastec Passive Diffusion tubes 1DL	Measuring time: 0.5–24 h	none	Passive	Integrated	No		Color Dosimetry	0.4–400 LOD 0.2 ppm (24 h)
Enerac 100 Pocket	8	6 AA rechargeable	Active		Yes	Yes	Electrochemical sensor	0–2,000
IAQ CALC indoor air quality meter 7545		4 AA AC Adapter	Passive	Continuous	Yes	Yes	Electrochemical sensor	0–500

The most commonly used biomarkers are CO levels in exhaled breath or carboxyhemoglobin measurements. Exhaled breath CO is a non-invasive measurement of the concentration of CO exhaled by a person. CO in the bloodstream attaches to hemoglobin to form carboxyhemoglobin, a stable complex measured through a blood test or non-invasively using a CO-oximeter. Although both exhaled breath CO and carboxyhemoglobin blood tests are accurate methods to quantify CO exposures, they only provide a snapshot of the current internal dose at the time of measurement. Caution should be used, as the measured value will vary based on the time between exposure and measurement. The internal dose decreases over time as the body metabolizes the CO, making it difficult to determine cumulative CO exposures over a specific time period or to compare measurements between study subjects (Eppler et al. 2013).

In HAP studies, CO has been measured as a proxy for exposure when personal exposure measurements of PM_2.5_ have not been feasible, for example, when monitoring very young children unable or unwilling to endure carrying gravimetric sampling equipment. The evidence for the adequacy of CO as a proxy for fine particulates is currently limited and inconclusive. Smith et al. (2011) measured CO exposure in children aged less than 18 months and determined the relationship between CO and PM as a proxy for PM concentrations in the world’s first randomized control trial with a cookstove intervention. However, more recent findings on the use of CO as a proxy for CO concentrations are varied. In Gambia (Dionisio et al. 2012), CO was found to be a poor proxy for personal exposures to PM. On the other hand, McCracken et al. (2013) reported strong evidence for the use of CO as a proxy for personal PM exposures in a longitudinal study of adult women in rural Guatemala. There are several limitations to using CO as a proxy for personal exposures to PM (Naeher et al. 2001; Northcross et al. 2010), and it is suggested that direct measurements of PM should be made where possible. However, CO will continue to serve as a useful indicator of exposure in cases where there is biological plausibility for CO-related mechanisms of health effects, such as adverse pregnancy outcomes (Thompson et al. 2011) and seizures, and in cases where a more imprecise measure of exposure to HAP is sufficient. There are advantages and limitations for both CO and PM monitors. Tables 2 and 3 summarize the characteristics of available instrumentation. Designing an exposure assessment plan requires balancing monitor cost, data handling logistics, monitor placement and size, length of required battery life, monitor accuracy, and required measurement ranges. Tables 2 and 3 summarize these properties for commercially available monitors; the best monitor for any given project will vary based on the needs of the study.

**Table 3:** Summary of Commercially Available Particulate Matter Monitors

Name	Monitor type	Battery type	Max battery life (h)	Active/passive	Integrated/continuous	Data logging	Zero correction	Detection method	PM cutpoint	Manufacturer detection limit/measurement range****
RTI MicroPEM	Personal	3 AA	≥36	Active	Integrated Continuous	Yes	Yes	Light scattering Gravimetric	PM_2.5_ PM_10_	LOD: 1 μg/m^3^
Berkeley Air UCB	Personal	9 V battery	~600	Passive	Continuous	Yes	Yes	Light scattering	none	LOD: 30–50 μg/m^3^ UOD ~25,000 μg/m^3^
TSI Side Pak AM510	Personal	Rechargeable 1,650 mAH 2,700 mAH NiMH 6-cell AA	7–22	Active	Continuous	Yes	Yes	Light scattering	PM_2.5_ PM_10_	PM size: 0.1–10 μm Res/: 1 μg/m^3^
DustTrak 2	Area	Rechargeable 6,600 mAH Li-Ion 3,600 mAH Li-Ion	<6	Active	Continuous	Yes	Yes	Light scattering	PM_2.5_ PM_10_	PM Size: 0.1–10 μm Mass Conc.: 0.001–150 mg/m^3^
MIE Personal DataRAM. pDR-1000AN	Personal	Rechargeable NiMH 9 V alkaline 9 V Li AC source	NiMH:72 9 V alkaline: 20 9 V Li: 40	Passive	Continuous	Yes	Yes	Light scattering	none	PM Size: 0.1–10 μm Mass Conc.: 0.001–400 mg/m^3^
MIE Personal DataRAM. pDR-1200	Personal	Rechargeable NiMH, or AC line current	NiMH:72 9 V alkaline: 20 9 V Li: 40	Active	Continuous	Yes	Yes	Light scattering	none	PM Size: 0.1–10 μm Mass Conc: 0.001–400 mg/m^3^
SKC Airchek model 224-PCXR8 Pump	Personal	Rechargeable NiMH	>20	Active	Integrated	Yes	No	Gravimetric filter	–	NA
SKC Personal Exposure monitors (PEM model 200)	Personal	None	NA	Active	Integrated	NA	No	Gravimetric filter	PM_2.5_ PM_10_	NA
Sioutas Impactor and Leland Legacy Pump	Area/Short-term Personal	Li-Ion battery pack	24	Active	Integrated	NA	NA	Gravimetric filter	PM_2.5_ PM_1.0_ PM_0.5_ PM_0.25_	NA
Casella Apex Personal Sampling Pump	Personal	4.8 NiMH, AC line current	>45	Active	Integrated	No	–	Gravimetric filter	–	NA

## Who to Monitor?

HAP is caused primarily by cooking and heating inside or around the home. Individuals who are among the most highly exposed can be determined by identifying those responsible for cooking and heating, as well as those who spend time inside the home during active cooking. For example, a household may include several adult women, with only one or two in charge of the majority of the cooking. Restricting a study to the primary cooks focuses on those most highly exposed to HAP. Another example would be to restrict a childhood pneumonia study to children young enough to be carried on their mothers’ backs. As children learn how to walk, they spend less time in close proximity to their mother and therefore reduce the time spent in the kitchen while cooking occurs. Other populations that have been overlooked by most studies to date include the elderly, men, and older children. The decision of who and where to monitor will be driven by the goals and resources of the study. Understanding the household dynamic of the population of interest is essential to determining the most appropriate participant for a given study.

## Where to Place Monitors?

Assessing exposure in HAP studies requires measuring the concentration of pollutants of interest as well as the time a person is exposed to that concentration. Concentrations of pollutants can be measured in a number of locations, ranging from biomarkers from blood or urine samples to air sampling on a person to indoor household area measurements.

Biomarkers of biomass exposure have the potential to improve epidemiological accuracy in exposure and health outcome studies. While air pollution monitors measure external concentrations, biomarkers reflect the internal dose of pollutants and may even be able to distinguish between multiple pollution sources (Mayeux 2004; Rylance et al. 2013). Urine and blood serum samples are the most commonly studied biomarkers in HAP exposure studies, however, no cost-effective and validated biomarkers for HAP are currently available for field-use (Rylance et al. 2013).

With the exception of biomarkers, personal exposure measurements provide the most direct measure of pollutant exposure for an individual. Placing a monitor on a subject for a specified period of time gives an accurate measurement of pollutants in the air directly surrounding the participant. Personal monitoring requires monitors that are small, light, and quiet enough to be carried by the participant. Minimizing discomfort caused by personal monitoring is particularly challenging when studying infants and young children.

On a related note, compliance is extremely important to achieving accurate exposure measurements, and can be improved by pilot testing monitoring protocols in the study population. The most convenient location to carry the planned monitors (at the waist, on the back, in the breathing zone, etc.), the preferred type of carrying bag, and the maximum amount of time volunteers are willing to carry the equipment are all important factors to consider during a preliminary test. It is also ideal to assess participant compliance during the actual study through methods by using data logging accelerometers in conjunction with the monitors or asking participants questions about carrying the monitor. In addition to ensuring compliance, it is critical to ensure that wearing a monitor causes minimal disruption to participants’ daily routines. For example, if a woman in a study is embarrassed to leave the house while wearing the monitoring equipment, the measured HAP exposures may be larger than her typical exposures. It may be difficult to ascertain these issues, but they can be minimized by verifying the wear-ability and likability of the monitoring equipment.

When personal exposure monitoring is not a viable option, micro-environmental area-based monitoring can be conducted as an alternative. Micro-environmental monitoring relies on taking concentration measurements in areas where the person of interest spends time. Depending on the goal of the study, measurements can be taken from a single location, usually the kitchen, or in several locations throughout the house. Area-based measurements are not as accurate as personal monitoring since individual exposure depends on the proximity to the HAP source and the length of time exposed. Pollution concentrations can vary throughout areas, and the measured concentration is highly dependent on the placement of the monitor. A significant amount of spatial variability can occur within a specified area and care should be taken to determine the location and elevation which best represents the concentrations or exposures of interest. For example, if the measurements are to represent the personal exposure of the cook, then a monitor may be placed near the stove at the height of the cook. Area concentration ‘measurements can be used as a proxy for personal exposures, or as a part of a time-location-based exposure model developed to estimate personal exposures y. Area-based kitchen level measurements have been shown to provide a reasonable estimate of group-level differences; however, they may not perform as well as direct estimates of individual level personal exposures (Dionisio et al. 2012). Improvements in personal exposure estimates based on area level measurements can made using information about the amount of time spent in the same area as the monitor. Area-based measurements can also be used to quantify household level exposures.

Although the kitchen is commonly the largest source of PM_2.5_ and CO when solid fuels or inefficient cookstoves are used, it is important to determine if there are other possible sources large enough to impact exposures measurements. Examples include fires made outside of the home for cooking animal food, burning refuse, heating water, or for occupational purposes. All secondary sources that are used regularly should be included in the exposure assessment plan either by directly measuring concentrations or, at a minimum, acknowledging the source and its potential influence on the data.

## How to Monitor?

### Instrument Requirements and Options

Instrumentation requirements to conduct exposure assessment of household air pollution are similar for both CO and PM_2.5_. The need to measure across the large range of concentrations (1 ppm for CO and 1 μg/m^3^ for PM_2.5_ to 2,000 ppm for CO and 100 mg/m^3^ for PM_2.5_) with a resolution of 1 ppm or μg/m^3^ can present a challenge. Using monitors that do not meet these standards can result in under- or over-estimations. Tables 2 and 3 summarize the currently available monitoring instrumentation for PM_2.5_ and CO that are appropriate for HAP studies.

### Continuous Versus Integrated Sampling

Continuous sampling allows a researcher to measure concentrations of a pollutant over time and provides the flexibility to categorize data into time periods of interest. Most continuous monitors can measure at a time resolution of at least 60 s, with some resolved to single second intervals. Using multiple continuous monitors can also provide insight into the relationship between exposures to different pollutants with respect to time, frequency of elevated exposures, as well as maximum and minimum concentrations of exposure. Integrated monitors collect a sample over a predetermined amount of time. Cookstove studies focused on health outcomes are typically interested in at least 24-h averages, although shorter ‘peak’ time periods, for instance a meal time, can also produce useful information for cookstove intervention evaluation projects.

### Instrument Power

Power requirements for instrumentation vary based on the placement of the monitor as well as on access to electricity in the study location. If personal monitoring requires subjects to wear monitors, battery-operated monitors will be required. Battery-powered instruments are also required for studies in resource-limited areas where homes lack reliable electricity. Ideally, all battery-powered instruments used for a 24-h measurement should be able to run continuously for a minimum of 25 h. If longer sampling periods are required, batteries can either be changed in the field or continuous intermittent sampling can be used. This allows a monitor to operate on a preset timer (e.g., 1 min on 1 min off) and extends the battery life. It is essential to test the battery life of a monitor in field conditions. Manufacturers report battery life under conditions of normal use, which may not represent conditions found in HAP studies.

### Software and Data Management

Data analysis and management of integrated measurements should be considered during the study’s planning phase. Continuous monitors produce large numbers of data points (eg., One monitor sampling at a 1 min resolution for 24 h creates 1,440 data points), and when multiplied by each participant, the amount of data quickly increases. Automatic data cleaning and analyses using computer programming can save time and ensure consistency with larger datasets.

### Monitor Cost Versus Measurement Cost

Selection of HAP equipment for a study is almost always impacted by cost. The cost of the monitor is only a portion of the cost per sample. Additional costs can include equipment required for calibration (e.g., span gas, primary flow calibrators), as well as personnel costs necessary for sample preparation (e.g., filter weighing, tube labeling, monitor pre-testing), data analysis, data management, and equipment servicing. All associated costs should be considered when planning a study before making decisions about the type of monitor to purchase.

## When to Monitor?

Determining the frequency, length, and time of the sampling period is a crucial component of any study conducting an exposure assessment. Exposure to HAP from cookstoves can vary by season, day of the week, and time of day. Temporal effects can be caused by environmental factors such as season, temperature and precipitation; behavioral factors based on culture or the environment; as well as biological changes, especially in the case of growing children. These factors should be accounted for when conducting an exposure assessment.

The relationship between exposure to HAP and the health effect of interest should also be considered when designing a sampling plan. For example, it is important to monitor exposures during pregnancy for a birth outcome study. When the time window between exposure and health outcome is unknown, it may be necessary to conduct monitoring over a longer period. In studies that require lifelong exposures, it may be necessary to make informed estimates for previously occurring exposures.

Many studies have used 24- or 48-h monitoring periods to determine the average daily concentration of HAP. It is then assumed that these one-time exposure estimates are representative of time periods lasting anywhere from 1 week to 1 year. The ideal method to determine the appropriate sampling time is to use previously collected data on the inter- and intra-household variability of HAP concentrations. Knowledge of the variability of HAP concentrations over days, months, and seasons can inform the design of an ideal sampling plan for exposure assessment. Data on the variability of exposure can also be used to estimate the precision of using a single measurement to represent average exposures over a longer period of time.

McCracken et al. (2009) investigated the variance of personal exposure measurements within a cookstove intervention trial in the highlands of Guatemala. Forty-eight hour personal exposure measurements to CO were taken every 3 months for the first 18 months of life for 515 children. The results showed that collecting a greater number of repeated measures per subject may increase precision. This study also found that group-level (intervention vs control) estimates were better at predicting long-term (18 months) exposure using measured subject characteristics to explain between-subject variation.

## Future Technological Developments

Health studies have already illuminated the health damaging potential of smoke from solid fuel used for cooking. Although there is more to learn about the health impacts of exposure to cookstove smoke, the world is moving forward to reduce exposures by scaling up adoption of clean cookstoves and the use of cleaner fuels, including ethanol, LPG, biogas, and electricity. Future studies aimed at demonstrating the potential health benefits of adopting clean cooking technologies will be aided by improvements in measurement tools. In this effort, monitoring and evaluation of both stove usage and performance will be essential to achieve the greatest results, since *“You don’t get what you expect. You get what you inspect”* (Kirk R. Smith). The development of simple, low cost methods and tools will support future public health research and evaluation of stove implementation programs.

The development of small particle monitoring devices that can measure at the ultra-fine level and/or quantify black carbon content will be very useful to determine the in-field performance of new stove technologies and their impact on exposures to pollutants beyond CO and PM. For example, a new stove may significantly reduce exposures to PM_2.5_ mass, but increase exposure to ultra-fine particulate mass or black carbon. It is currently not clear how changes in exposures to these different PM components would affect health. In addition to being able to monitor personal exposures to different particle size fractions, or compositions, reducing the cost of sensors and monitors will further enable more exposure assessments with increased resolution in health studies. In particular, the development of compact, low cost, integrated monitors and sensors for CO, VOCs, particles, temperature, and geographic coordinates will increase the ability to monitor more subjects, or monitor for multiple sampling periods, both of which can reduce exposure measurement error. The development of low cost, low power consumption, wireless data transfer technology provides a major breakthrough in field operations, since wireless data transfer eliminates the need to visit each monitoring site, thus reducing cost and required personnel. In the near future, it is hoped that wireless data transfer of stove use monitors, air quality data, and stove performance data will be able to provide valuable information on the real-life effectiveness of stove intervention programs, as well as their potential benefits to public health.

## Capacity Building

As the world moves forward with scaling up cleaner cooking technologies, there is also an urgent need to build technical capacity in monitoring and evaluation, exposure assessment, and stove performance evaluation. The goal of capacity development should not be limited to supportive roles of researchers from developing countries while researchers from economically resource-rich countries take the lead. Capacity building should be aimed at ensuring self-sufficiency and development of world-class researchers and program implementation experts from the countries where cookstove interventions are occurring. For example, expanding the Global Alliance’s approach to developing in-country regional testing centers to include field-based exposure assessment will be a valuable contribution. Capacity needs include skills development for personnel as well as increased access to the latest monitoring equipment. The intrinsic cultural knowledge possessed by in-country researchers is invaluable and has been limited or lacking in earlier studies. Having access to the needed funding, equipment, and skills to study the health damaging effects of exposure to smoke from cooking with solid fuels not only empowers researchers from resource-limited countries, but can also highlight country- or region-specific issues.

## References

[CR1] Astrup P, Trolle D, Olsen HM, Kjeldsen K (1972). Effect of moderate carbon-monoxide exposure on fetal development. The Lancet.

[CR2] Aunan K, Alnes LWH, Berger J, Dong Z, Ma L, Mestl HES (2013). Upgrading to cleaner household stoves and reducing chronic obstructive pulmonary disease among women in rural China—a cost-benefit analysis. Energy, Sustainable Development.

[CR3] Banerjee A, Mondal NK, Das D, Ray MR (2012). Neutrophilic inflammatory response and oxidative stress in premenopausal women chronically exposed to indoor air pollution from biomass burning. Inflammation.

[CR4] Brook RD, Rajagopalan S, Pope CA, Brook JR, Bhatnagar A, Diez-Roux AV (2010). Particulate matter air pollution and cardiovascular disease an update to the scientific statement from the American Heart Association. Circulation.

[CR28] CDC (2013) Health Effects Carbon Monoxide Poisoning Exposure and Risk—CDC Tracking Network. http://ephtracking.cdc.gov/showCoRisk.action

[CR5] Clark ML, Bazemore H, Reynolds SJ, Heiderscheidt JM, Conway S, Bachand AM (2011). A baseline evaluation of traditional cook stove smoke exposures and indicators of cardiovascular and respiratory health among Nicaraguan women. International Journal of Occupational and Environmental Health.

[CR6] Clark ML, Peel JL, Balakrishnan K, Breysse PN, Chillrud SN, Naeher LP, et al. (2013) Health and household air pollution from solid fuel use: the need for improved exposure assessment. *Environmental Health Perspectives* 121:1120–1128. doi:10.1289/ehp.1206429 10.1289/ehp.1206429 PMC3801460 23872398

[CR7] Commodore AA, Hartinger SM, Lanata CF, Mäusezahl D, Gil AI, Hall DB (2013). A pilot study characterizing real time exposures to particulate matter and carbon monoxide from cookstove related woodsmoke in rural Peru. Atmospheric Environment.

[CR8] Dionisio KL, Howie SRC, Dominici F, Fornace KM, Spengler JD, Adegbola RA (2012). Household concentrations and exposure of children to particulate matter from biomass fuels in the Gambia. Environmental Science and Technology.

[CR9] Dix-Cooper L, Eskenazi B, Romero C, Balmes J, Smith KR (2012). Neurodevelopmental performance among school age children in rural Guatemala is associated with prenatal and postnatal exposure to carbon monoxide, a marker for exposure to woodsmoke. NeuroToxicology.

[CR10] Eppler AR, Fitzgerald C, Dorner SC, Aguilar-Villalobos M, Rathbun SL, Adetona O (2013). Using exhaled carbon monoxide and carboxy-hemoglobin to evaluate the effectiveness of a chimney stove model in Peru. International Journal of Occupational and Environmental Health.

[CR11] Lim SS, Vos T, Flaxman AD, Danaei G, Shibuya K, Adair-Rohani H (2012). A comparative risk assessment of burden of disease and injury attributable to 67 risk factors and risk factor clusters in 21 regions, 1990-2010: a systematic analysis for the Global Burden of Disease Study 2010. The Lancet.

[CR12] Mayeux R (2004). Biomarkers: potential uses and limitations. NeuroRx.

[CR13] McCracken JP, Schwartz J, Bruce N, Mittleman M, Ryan LM, Smith KR (2009). Combining Individual- and Group-Level Exposure Information. Epidemiology.

[CR14] McCracken JP, Schwartz J, Diaz A, Bruce N, Smith KR (2013) Longitudinal relationship between Personal CO and Personal PM2.5 among women cooking with woodfired cookstoves in Guatemala. *PLoS ONE* 8:e55670. doi:10.1371/journal.pone.0055670 10.1371/journal.pone.0055670 PMC3582619 23468847

[CR15] Mukhopadhyay R, Sambandam S, Pillarisetti A, Jack D, Mukhopadhyay K, Balakrishnan K (2012). Cooking practices, air quality, and the acceptability of advanced cookstoves in Haryana, India: an exploratory study to inform large-scale interventions. Global Health Action 5.

[CR16] Naeher LP, Brauer M, Lipsett M, Zelikoff JT, Simpson CD, Koenig JQ (2007). Woodsmoke health effects: a review. Inhalation Toxicology.

[CR17] Naeher LP, Smith KR, Leaderer BP, Neufeld L, Mage DT (2001). Carbon monoxide as a tracer for assessing exposures to particulate matter in wood and gas cookstove households of Highland Guatemala. Environmental Science and Technology.

[CR18] Northcross A, Chowdhury Z, McCracken J, Canuz E, Smith KR (2010). Estimating personal PM2.5 exposures using CO measurements in Guatemalan households cooking with wood fuel. Journal of Environmental Monitoring.

[CR19] Pine K, Edwards R, Masera O, Schilmann A, Marrón-Mares A, Riojas-Rodríguez H (2011). Adoption and use of improved biomass stoves in Rural Mexico. Energy Sustainable Development.

[CR20] Rylance J, Gordon SB, Naeher LP, Patel A, Balmes JR, Adetona O, et al. (2013) Household air pollution: a call for studies into biomarkers of exposure and predictors of respiratory disease. *Amercan Journal of Physiology: Lung Cellular and Molecular Physiology* 304:L571–L578. doi:10.1152/ajplung.00416.2012 10.1152/ajplung.00416.2012 PMC3652022 23457186

[CR21] Shankar A, Johnson M, Kay E, Pannu R, Beltramo T, Derby E (2014). Maximizing the benefits of improved cookstoves: moving from acquisition to correct and consistent use. Global Health: Science and Practice.

[CR22] Sinton JE, Smith KR, Peabody JW, Yaping L, Xiliang Z, Edwards R (2004). An assessment of programs to promote improved household stoves in China. Energy Sustainable Development.

[CR23] Smith KR, McCracken JP, Weber MW, Hubbard A, Jenny A, Thompson LM (2011). Effect of reduction in household air pollution on childhood pneumonia in Guatemala (RESPIRE): a randomised controlled trial. The Lancet.

[CR24] Thompson LM, Bruce N, Eskenazi B, Diaz A, Pope D, Smith KR (2011). Impact of reduced maternal exposures to wood smoke from an introduced chimney stove on newborn birth weight in rural Guatemala. Environmental Health Perspectives.

[CR25] Venkataraman C, Sagar AD, Habib G, Lam N, Smith KR (2010). The Indian National Initiative for Advanced Biomass Cookstoves: the benefits of clean combustion. Energy Sustainable Development.

[CR26] World Health Organization (2006) *Air Quality Guidelines: Global Update 2005*. World Health Organization

[CR29] World Health Organization (2014) Ambient and household air pollution and health. http://www.who.int/phe/health_topics/outdoorair/databases/en/

[CR27] Yu O, Sheppard L, Lumley T, Koenig JQ, Shapiro GG (2000). Effects of ambient air pollution on symptoms of asthma in Seattle-area children enrolled in the CAMP study. Environmental Health Perspectives.

